# Adaptation of travel medicine practitioners to the COVID-19 pandemic: a cross-sectional survey of ISTM members

**DOI:** 10.1093/jtm/taac032

**Published:** 2022-03-03

**Authors:** Rebecca W Acosta, Jenny T Visser, Lin H Chen, Peter A Leggat, Gerard T Flaherty

**Affiliations:** Traveler’s Medical Service of New York, New York, NY, USA; Wellington School of Medicine, Wellington, New Zealand; Division of Infectious Diseases and Travel Medicine, Mount Auburn Hospital, Cambridge, MA, USA; Harvard Medical School, Boston, MA, USA; College of Public Health, Medical and Veterinary Sciences, James Cook University, Townsville, Australia; School of Medicine, National University of Ireland Galway, Galway, Ireland; School of Medicine, National University of Ireland Galway, Galway, Ireland; School of Medicine, International Medical University, Kuala Lumpur, Malaysia

## Abstract

This cross-sectional study evaluated the adaptations of current International Society of Travel Medicine (ISTM) members in relation to the disruption caused by the coronavirus disease 2019 (COVID-19) pandemic. It demonstrates that the majority of members remain engaged with travel medicine and ISTM educational activities, while adapting to COVID-related clinical demands.

The global pandemic of coronavirus disease 2019 (COVID-19) has severely compromised international travel and the practice of travel medicine. Uncertainty surrounds new procedures for travel and high levels of public anxiety around travelling are evident, especially with the emergence of further COVID-19 variants.^1^^,^^2^ Although there have been some insightful reflections in the recent literature,^3–5^ the impact on and the response of the travel medicine community to this pandemic have not been formally evaluated. The International Society of Travel Medicine (ISTM) represents the largest group of travel healthcare professionals. Our study aimed to evaluate the attitudes and adaptations of current ISTM members in relation to the disruption caused by the COVID-19 pandemic. The specific objectives of the study were to ascertain the extent to which members’ clinical practice has been affected by the pandemic and inform the Society’s strategic planning for upcoming activities and member engagement during the pandemic and early post-pandemic period.

This cross-sectional study involved the electronic distribution via the ISTM secretariat of an anonymous 27-item questionnaire created on Survey Monkey™, comprising a mixture of multiple-choice, ranked and free text responses. The questionnaire was developed and piloted in consultation with the ISTM Leadership Council, which includes the chairpersons of its committees, professional groups and interest groups. The ISTM membership received fortnightly invitations to participate. The survey closed after 2 months (April–May 2021) and the results were downloaded to a Microsoft Excel (2016) database, which was used to descriptively analyse the aggregated data, using frequencies and percentages. Institutional review board approval was received from the clinical research ethics committee of Galway University Hospitals (C.A. 2584).

A total of 227 (10.9%) of the 2093 current ISTM members responded to the survey. The majority of respondents were physicians (63.8%, *n* = 118), followed by nurses (15.7%, *n* = 29) and pharmacists (8.1%, *n* = 15). A majority of respondents were based in North America (53.8%, *n* = 118), Europe (17.8%, *n* = 33) and Oceania (8.7%, *n* = 16). The respondents closely reflected the demographics of the wider membership base. The principal affiliations of most respondents were academic institutions (23.8%, *n* = 44), private travel clinics (22.2%, *n* = 41), general medical practices (22.2%, *n* = 42) and hospitals (9.2%, *n* = 17). The majority of respondents (72.6%, *n* = 135) had been ISTM members for at least 6 years. More than one-half (58.2%, *n* = 107) had earned the ISTM Certificate in Travel Health™ credential.

**Table 1:** Thematic summary of qualitative data arising from ISTM member survey

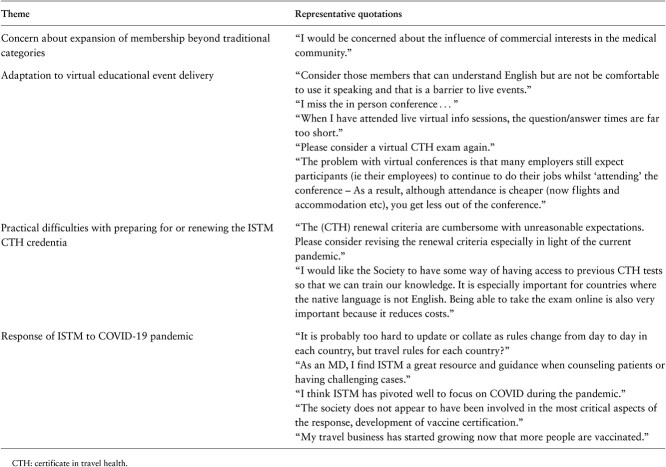

Although most respondents (77.5%, *n* = 176) were actively engaged in travel medicine throughout the pandemic, they had experienced a substantial reduction in the volume of their travel clinical practice, with 70.2% (*n* = 106) reporting that travel medicine now comprised <25% of their practice. A minority (18.4%, *n* = 34) had pivoted to other areas of clinical activity, 13% (*n* = 24) had been redeployed to other areas of healthcare, whereas 1.1% (*n* = 2) opted to leave travel medicine practice. The most common COVID-related clinical activities in this cohort of ISTM members were testing and screening for COVID-19 (55.7%, *n* = 103), providing COVID-19 vaccine information (53%, *n* = 98) and administering COVID-19 vaccines (42.7%, *n* = 79). Over 90% (92.93%, *n* = 171) of respondents had already received or planned to receive a COVID-19 vaccine and 91.35% (*n* = 169) would encourage their patients to be vaccinated against the disease. The majority of respondents were either likely (66.7%, *n* = 124) or somewhat likely (20.4%, *n* = 38) to practise travel medicine in a post-pandemic setting.

The majority (96.8%, *n* = 180) indicated that they intended to renew their ISTM membership. The most commonly reported motivation for joining ISTM (95.7%, *n* = 176) was to become part of a community of likeminded professionals. The majority (60%, *n* = 111) expressed a wish to become involved in travel health research. There was a very high level of satisfaction with the educational services provided by ISTM. The COVID-19-related ISTM resources, which were deemed most useful were webinars (54.1%, *n* = 99), *Journal of Travel Medicine* collections (47%, *n* = 86) and *TravelMed* listserv discussion posts (42.7%, *n* = 78). The preferred mode of delivery of educational material was the in-person medical conference (94%, *n* = 172), followed by web-based journals or e-books (90.7%, *n* = 166) and webinars (88%, *n* = 161). The majority (88.1%, *n* = 162) of respondents expressed a desire to learn more about COVID-19 and travel, whereas 65.4% (*n* = 121) reported that they would like to learn more about the use of social media in travel medicine.

Nearly two-thirds of respondents (65.4%, *n* = 119) expressed an interest in participating in meet-the-expert sessions; 64.7% (*n* = 119) were interested in intermittent access to a senior practitioner for clinical advice; whereas 53.6% (*n* = 98) stated that they would like to participate in an informal question and answer panel. Only 35.5% (*n* = 65) of respondents were interested in a mentoring program. Respondents were largely positive about the possibility of ISTM expanding its membership options to include non-traditional members, with two-thirds being very open (45.4%, *n* = 84) or somewhat open (21.6%, *n* = 40) to the proposal. Pharmacists and members from under-represented geographic regions had a more favourable response to the expansion of ISTM membership beyond its traditional professional categories. Thirty-three free text responses were received, which were analysed independently by three researchers (RWA, JTV and GTF). Qualitative data are summarized thematically in Table 1.

As the number of international travellers fell dramatically in response to the COVID-19 pandemic, the global travel and tourism industry suffered significant losses. Given the transmission characteristics of severe acute respiratory syndrome coronavirus 2 (SARS-CoV-2) and its cross-border spread,^6^ there has been an intense focus in the medical literature on the safety of air travel in the transmission of SARS-CoV-2.^7^ The counselling of travellers about prevention of COVID-19 and entry requirements to cross international borders have been routinely integrated into pre-travel consultations.^8^ Screening for infection with or immunity against SARS-CoV-2 has now become an additional clinical service offered by many travel clinics.^9^

Our cross-sectional study demonstrates that the majority of survey respondents remain engaged with travel medicine, while adapting to COVID-related activities, including the rollout of their national COVID-19 vaccination programs and providing COVID testing and clinical services. In addition, the seventeenth Conference of the ISTM (CISTM17) successfully pivoted to an immersive virtual platform, which was very well attended by both members and non-members.^10^ Although in-person conferences are the preferred educational medium for most members, there is an appreciation for the need and flexibility of digital educational events and resources, especially webinars and social media, to continue to be part of the offering, even in a post-pandemic era. Novel learning activities that provide opportunities for closer interaction with travel medicine experts also appear to be highly valued.

The challenges of transforming to a more agile organization, capable of responding more effectively to its members’ learning needs by applying innovative digital technology while preserving the cherished in-person contact between members are reflected in the respondents’ free text comments. There is a need for suitably designed qualitative studies to probe these and other member concerns more deeply, however. We also advocate that research into ISTM members’ views about the future of travel medicine, emerging international travel patterns, the role and applications of technology such as teleconsultations in travel medicine practice and how the pandemic has uniquely affected special groups of travellers and influenced travel hygiene awareness, be prioritized. Longitudinal follow-up member surveys may allow a clearer picture to emerge of the full extent to which COVID-19 has affected travel medicine practice and activity as we enter a new phase of the pandemic.

This study was limited by its response rate, which may have been related to its administration at a time of heightened global activity, with the spread of the delta variant of COVID-19 and members’ shift of focus to pandemic response and other professional activities during a time of significant decrease in international travel. The professional and geographic distribution of respondents closely matches that of the ISTM as a whole, however. It is possible that some ISTM members who are not clinically active or who are retired from clinical practice did not complete the questionnaire. ISTM members who had not renewed their membership during the pandemic were also not likely to have been captured by this study. Although we cannot exclude volunteer bias, we are satisfied that the survey provides useful insights into the prevailing views and experiences of a cohort of experienced travel medicine providers and will help to inform society-level activities and research priorities as we continue to examine the impact of the pandemic on travel health practice and safe and healthy international travel.
